# No compelling evidence of sex differences in brain maturation during COVID-19 lockdowns when the sexes are compared statistically

**DOI:** 10.1073/pnas.2421462122

**Published:** 2025-04-01

**Authors:** Andrew W. Brown, Simon Chung, Timothy Koscik, Colby J. Vorland, Donna L. Maney

**Affiliations:** ^a^Department of Biostatistics, University of Arkansas for Medical Sciences, Little Rock, AR 72205; ^b^Arkansas Children’s Research Institute, Little Rock, AR 72202; ^c^Department of Pediatrics, University of Arkansas for Medical Sciences, Little Rock, AR 72205; ^d^Department of Epidemiology and Biostatistics, Indiana University School of Public Health-Bloomington, Bloomington, IN 47403; ^e^Department of Psychology, Emory University, Atlanta, GA 30322

Consideration of sex as a variable is increasingly regarded as important for replicability and generalizability of results (1). To maximize rigor, sex must be incorporated using valid statistical approaches. We were therefore concerned that Corrigan et al. (2) reported sex differences that were not tested statistically. The authors claimed that Covid-19-related lockdowns had a “more severe impact on the female brain than the male brain,” showing “the greater vulnerability of the female brain, as compared to the male brain” when they did not compare the sexes using valid methods. Here, by conducting the appropriate tests, we show that their published data do not support their claims of widespread sex differences.

Instead of comparing the sexes statistically to support their claims, Corrigan et al. tested for effects within each sex separately, declaring a sex difference when the null hypothesis was rejected in one sex but not the other. This approach has, for decades, been widely recognized as invalid (3–6). When applied to sex differences, it has been called the “Difference in Sex-Specific Significance” (DISS) error (7, 8), which is a specific case of the “Differences in Nominal Significance” (DINS) error (7). When considering two groups (as is often the case when considering sex as a variable), DISS can result in false positive findings of difference up to 50% of the time—no better than flipping a coin (9).

Using a DISS approach, Corrigan et al. reported two main findings. First, they reported that cortical thinning deviated significantly from normative values in 30 regions of the “female brain” and in two regions of the “male brain.” Second, they reported a “difference” in overall age acceleration, based on their figure 5, which showed a CI crossing zero in males but not females. But neither of these findings are based on valid, direct comparisons between females and males. Discordant assessments of statistical significance do not inform or imply significant differences between sexes (3–6).

**Fig. 1.**
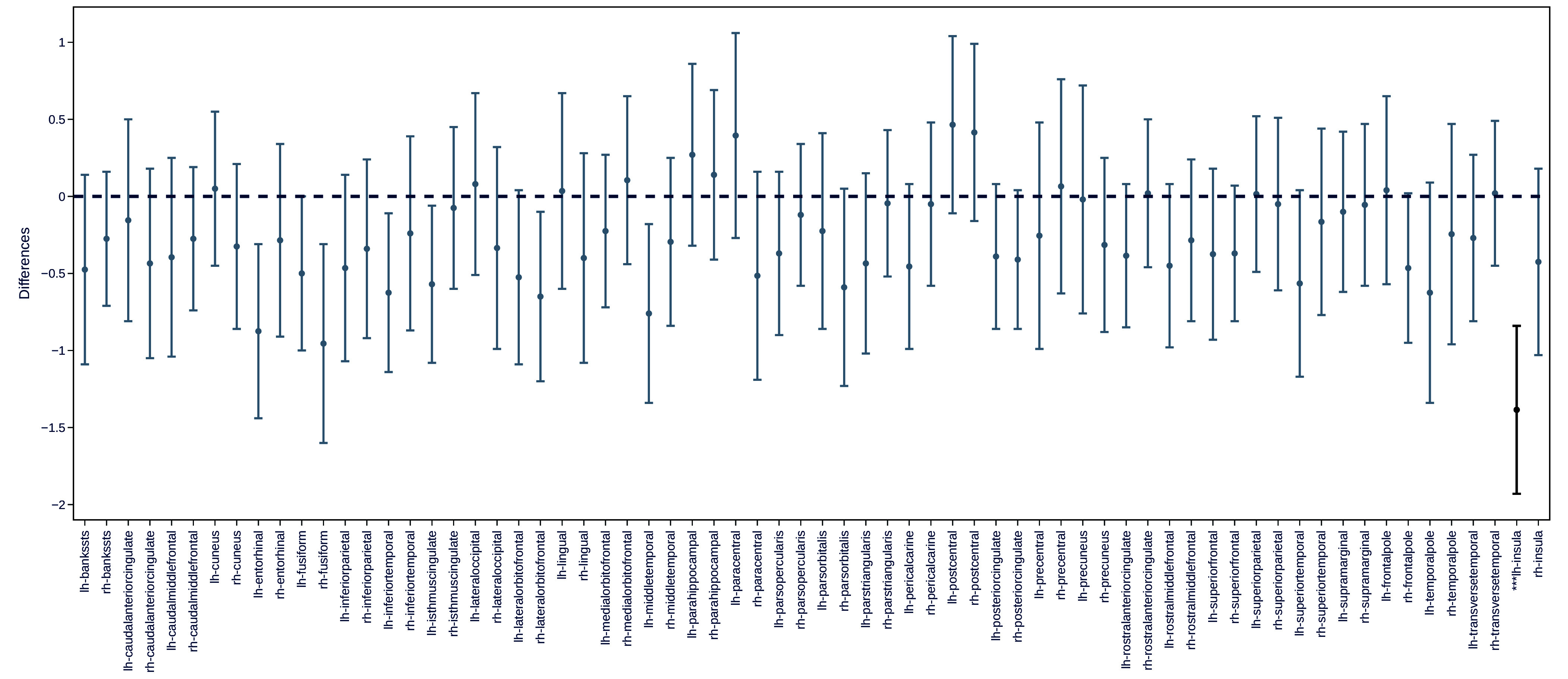
Only one of thirty sex differences in cortical thinning reported by Corrigan et al. (2) held up in a valid between-sex comparison. Point estimates represent the difference between males and females for each of 68 regions (named on the x-axis) with uncorrected 95% CI (compare with Corrigan et al., figure 4). After applying false discovery rate (FDR) correction as per the authors, only the difference in the left hemisphere insula (lh-insula, darker line on figure) remained significant. *P = 0.0004.

**Fig. 2.**
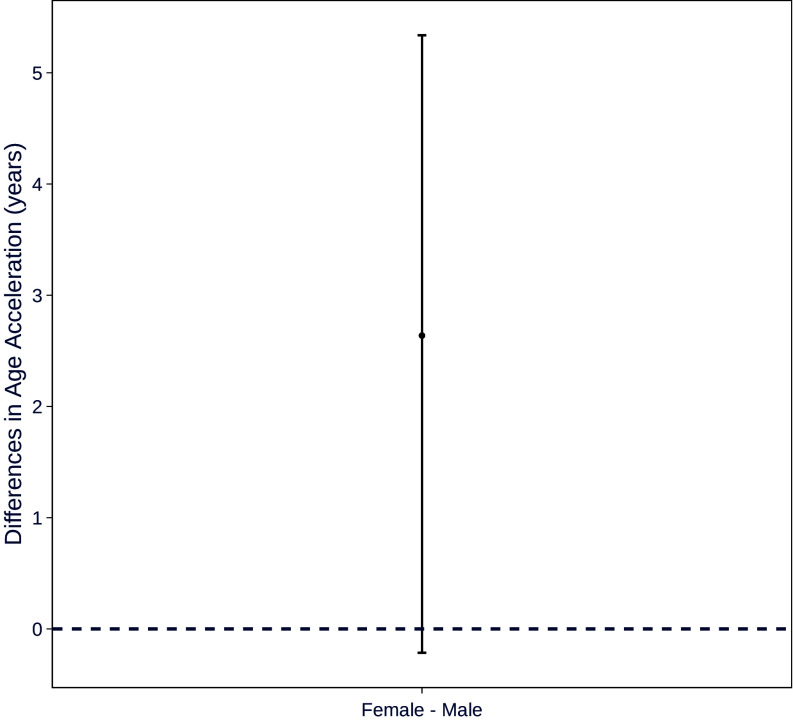
When comparing estimated age acceleration between the sexes statistically, the 95% CI includes the null. Compare with Corrigan et al., figure 5, in which a DISS approach was employed as evidence of significant age acceleration in females but not males.

We tested the authors’ claims about sex differences using their shared data, code, and normed region values. For each of 68 regions, we compared normative deviation estimates between females and males using two-sample *t* tests and the same false discovery rate correction used by Corrigan et al. Our analysis showed that cortical thinning was accelerated significantly more in females than males in only 1 region, not 30 (Fig. 1). In the case of overall “age acceleration,” the bootstrapped 95% CI failed to exclude the null (Fig. 2), meaning that, based on the authors’ threshold of statistical significance, the data do not convincingly support the claim of a sex difference.

Setting aside other limitations of the study, such as the sample size and how normative brain aging was estimated (10), our analysis shows little compelling evidence of the sex differences claimed by Corrigan et al. We emphasize that our analysis was possible only because of thorough and transparent reporting of data and code, for which the authors are commended.
